# Genome Sequence of the *Pectobacterium atrosepticum* Strain CFBP6276, Causing Blackleg and Soft Rot Diseases on Potato Plants and Tubers

**DOI:** 10.1128/genomeA.00374-13

**Published:** 2013-06-20

**Authors:** Anthony Kwasiborski, Samuel Mondy, Amélie Beury-Cirou, Denis Faure

**Affiliations:** Institut des Sciences du Végétal, CNRS, Gif-sur-Yvette, Francea; Comité Nord Plant de Pomme de Terre (CNPPT), Semences, Innovation, Protection Recherche et Environnement (SIPRE), Achicourt, Franceb

## Abstract

*Pectobacterium atrosepticum* strain CFBP6276 is a pectinolytic enterobacterium causing blackleg and soft rot of the stem and tuber of *Solanum tuberosum*. Its virulence is under the control of quorum sensing, with *N*-acylhomoserine lactones as communication signals. Here, we report the genome sequence of *P*. *atrosepticum* strain CFBP6276.

## GENOME ANNOUNCEMENT

Soft rot and blackleg diseases cause severe economic losses in potato production. Plant symptoms are characterized by black rot of the stem and rotting of potato tubers in the field, as well as in postharvest (1). Annual surveys conducted on potato cultures in France have shown that the *Pectobacterium* genus has been predominant as a cause of these diseases for >10 years (2). *Pectobacterium atrosepticum* CFBP6276 (99-16 in the FN3PT collection, France) was isolated by Valérie Hélias (FN3PT) from blackleg symptoms of *Solanum tuberosum* in France in 1999. In *P. atrosepticum* CFBP6276, quorum sensing (QS) is required for the expression of virulence factors, including pectinolytic enzymes and harpins (3–8). QS-mediated virulence of strain CFBP6276 may be attenuated by bacteria and enzymes that are able to inactivate QS signals (6, 8–10). Despite the worldwide occurrence of *P. atrosepticum* as a potato pathogen, a single whole-sequenced genome is available at this time, that of *P. atrosepticum* strain SCRI1043 (ATCC BAA-672). This strain was isolated from a blackleg potato stem in 1985 in Scotland and produces *N*-3-oxo-hexanoylhomoserine lactone as a main QS signal (11), while most other strains of *P. atrosepticum*, including CFBP6276, produce *N*-3-oxo-octanoylhomoserine lactone (12).

Here, we report the *de novo* genome assembly of *P. atrosepticum* strain CFBP6276. Two libraries were constructed using the TruSeq SBS v3 sequencing kit: a shotgun paired-end library with a fragment size between 150 and 500 bp and a long jumping distance mate-pair library with an insert size average of 6,000 bp. Libraries were sequenced using the 2 × 100 bp paired-end read module of Illumina HiSeq 2000 by Eurofins Genomics (France). Sequences reads with low-quality (<0.05) ambiguous nucleotides (*n* > 2) and sequence lengths of <30 nucleotides were discarded for the assembly. A total of 49,815,601 paired-end reads were retained (4,533,219,691 bases), with an average length of 91 bp, and 5,196,425 mate-paired reads (387,133,662 bases), with an average length of 74.5 bp. Sequence assembly was carried out using the CLC Genomics Workbench v5.1 (CLC bio, Aarhus, Denmark) with a read length of  0.5 and a similarity of  0.8 as parameters. The scaffolding was processed using SSPACE basic v2.0 (13). The *in-silico* finishing of some gaps was carried out by mapping (read length of 0.9 and similarity of 0.95) the mate-pair reads on each of the 5-kbp contig ends. Then, the collected reads were used for *de novo* local assembling (read length of 0.5 and similarity of 0.8). Some additional gaps were closed by Sanger sequencing of the PCR amplicons. The published sequence is composed of 9 contigs with a sequence length from 2 kbp to 1.9 Mbp, grouped in 4 scaffolds.

The *P. atrosepticum* CFBP6276 genome consists of one circular chromosome, containing 4,860,851 bp, and two circular plasmids, containing 5,876 bp and 2,681 bp, respectively. The percentages of G+C content are 51% for the chromosome and 47% for the plasmids. A total of 4,430 coding sequences were predicted using the Rapid Annotations using Subsystems Technology (RAST) v4.0 automated pipeline (14).

### Nucleotide sequence accession number.

The *P. atrosepticum* strain CFBP6276 genome sequence has been deposited in GenBank under the accession no. ASAB00000000.
